# Optimal Multichannel Artifact Prediction and Removal for Neural Stimulation and Brain Machine Interfaces

**DOI:** 10.3389/fnins.2020.00709

**Published:** 2020-07-17

**Authors:** Mina Sadeghi Najafabadi, Longtu Chen, Kelsey Dutta, Ashley Norris, Bin Feng, Jan W. H. Schnupp, Nicole Rosskothen-Kuhl, Heather L. Read, Monty A. Escabí

**Affiliations:** ^1^Department of Electrical and Computer Engineering, University of Connecticut, Storrs, CT, United States; ^2^Department of Biomedical Engineering, University of Connecticut, Storrs, CT, United States; ^3^Department of Biomedical Sciences, City University of Hong Kong, Kowloon, Hong Kong; ^4^Shenzhen Research Institute, City University of Hong Kong, Shenzhen, China; ^5^Neurobiological Research Laboratory, Section for Clinical and Experimental Otology, University Medical Center Freiburg, Freiburg im Breisgau, Germany; ^6^Department of Psychology, University of Connecticut, Storrs, CT, United States; ^7^The Connecticut Institute for the Brain and Cognitive Sciences, University of Connecticut, Storrs, CT, United States

**Keywords:** artifact removal, electrical stimulation, nerve fibers, cochlear implants, neural implant, brain machine interface, Wiener filter

## Abstract

Neural implants that deliver multi-site electrical stimulation to the nervous systems are no longer the last resort but routine treatment options for various neurological disorders. Multi-site electrical stimulation is also widely used to study nervous system function and neural circuit transformations. These technologies increasingly demand dynamic electrical stimulation and closed-loop feedback control for real-time assessment of neural function, which is technically challenging since stimulus-evoked artifacts overwhelm the small neural signals of interest. We report a novel and versatile artifact removal method that can be applied in a variety of settings, from single- to multi-site stimulation and recording and for current waveforms of arbitrary shape and size. The method capitalizes on linear electrical coupling between stimulating currents and recording artifacts, which allows us to estimate a multi-channel linear Wiener filter to predict and subsequently remove artifacts via subtraction. We confirm and verify the linearity assumption and demonstrate feasibility in a variety of recording modalities, including *in vitro* sciatic nerve stimulation, bilateral cochlear implant stimulation, and multi-channel stimulation and recording between the auditory midbrain and cortex. We demonstrate a vast enhancement in the recording quality with a typical artifact reduction of 25−40 dB. The method is efficient and can be scaled to arbitrary number of stimulus and recording sites, making it ideal for applications in large-scale arrays, closed-loop implants, and high-resolution multi-channel brain-machine interfaces.

## Introduction

Advances in neural implant and electrical stimulation technologies, such as cochlear implants (CIs) and vagal nerve stimulators, increasingly rely on concurrent neural stimulation and recordings to either assess functional transformations between connected brain regions (Lim and Anderson, 2007; Kral et al., 2009; Atencio et al., 2014; Hancock et al., 2017; Vollmer, 2018; Li et al., 2019) or to optimize electrical stimulation via closed-loop feedback control (Wilson et al., 1991; Schachter and Saper, 1998; Dhillon and Horch, 2005; Lebedev and Nicolelis, 2006; O’Doherty et al., 2011; Mc Laughlin et al., 2012; Hartmann et al., 2014). In such applications, capacitive and inductive coupling between the stimulating and recording electrodes leads to stimulus-evoked artifacts in the extracellular recordings that are often several orders of magnitude larger (i.e., milli-volts) than the extracellular neural signals (typically micro-volts). Such artifacts obscure neural activity and make it difficult to interpret and quantify neural data. Electrical stimulation artifacts are also present in surface recordings of electroencephalogram (EEG) and electrocorticogram (ECoG) that are more widely used clinically with concurrent functional neural stimulations in recent brain-machine interfaces and prosthetic devices (Peper and Grimbergen, 1983; Groves and Brown, 2005; Allen et al., 2010; Hoffmann et al., 2011). In order to obtain neural responses to electrical stimuli in the above recording situations, artifact removal is necessary to isolate neural signals for robust and high-fidelity assessment of neural activity. Such artifact removal will also be essential for real-time closed-loop stimulation in the next-generation of neural implants, brain machine interfaces, and prosthetic devices.

Existing artifact removal algorithms invariably focus on the recorded artifact waveforms without explicitly considering the stimulus currents and sources that are responsible for generating the artifacts. That is, most artifact removal algorithms do not explicitly use the electrical stimulation current waveforms to either predict or remove artifacts (although see Trebaul et al., 2016). Such techniques include artifact template subtraction (Wichmann, 2000; Hashimoto et al., 2002; Trebaul et al., 2016; Qian et al., 2017), local curve fitting (Wagenaar and Potter, 2002), sample-and-interpolate technique (Heffer and Fallon, 2008), and independent component analysis (Makeig et al., 1996; Vigário, 1997; Gilley et al., 2006; LeVan et al., 2006; Debener et al., 2008; Rogasch et al., 2014). Such artifact waveform-based algorithms usually estimate artifacts by statistical analysis of the recorded signals, which can suppress artifacts in certain stimulation/recording paradigms. A general assumption of these algorithms is that the recorded artifacts arise from single isolated stimulation sources that are reproducible and non-overlapping over time. This assumption may be valid for classic neural implants like the cardiac pacemaker but can deviate greatly from situations in advanced neural devices that utilize multichannel stimulus electrodes (i.e., multiple sources) and arbitrary stimulation waveforms with dynamically varying current amplitude, stimulation rate and/or pattern (e.g., as for cochlear implants). Further, such artifact removal techniques are often difficult to implement in real-time especially with dynamically varying stimulus paradigms. Instead, they are mostly used for *post hoc* removal of artifacts. To address the challenge of multi-site stimulation and recording, a recent method used advanced statistical modeling to improve spike-sorting quality from extracellular multi-channel recordings (Mena et al., 2017). Like other approaches, this method places assumptions on the statistical structure of the artifacts and neural waveforms that may not strictly hold and likewise does not directly use the known electrical stimulation signals to remove the artifacts. In addition, many waveform-based algorithms fail when multiple artifacts are generated in close succession during fast current stimulation. For example, cochlear implants (CIs) generate hundreds to thousands of stimulus pulses per second of varying amplitudes across multiple stimulation electrodes that often overlap in time (Friesen and Picton, 2010), a situation that challenges all current waveform-based artifact removal algorithms. One solution to enhance artifact removal in such scenarios is to decrease the rate of CI stimulation and use constant current amplitudes, which leads to abnormal stimulation scenarios that make it difficult to characterize normal stimulation and neural processing with such devices (Friesen and Picton, 2010).

Here, we develop an optimal multichannel artifact removal algorithm that can be applied during high-throughput multi-site electrical stimulation with arbitrary stimulation waveforms. Unlike nearly all other artifact removal algorithms, which are *blind* to the stimulation currents (i.e., the algorithm does not explicitly utilize the input current waveforms to predict or remove the artifacts), our method capitalizes on the fact that transformation between electrical stimulation currents and artifacts on the recording arrays arises through *linear* capacitive and inductive coupling (Rivnay et al., 2017) and the fact that stimulation currents are actually known *a priori* in most instances. We approach the artifact removal by first establishing the assumption that recorded artifacts behave linearly with respect to the stimulation currents. This allows us to derive optimal linear filters to model the transformation between each stimulating-recording electrode pair. The linear transfer functions for each stimulation and recording site are estimated as a digital filter, i.e., the Wiener filter, and can be updated as required during the recording procedure to track the adaptive changes in electrical coupling over time (due to long-term change in impedance, electrode movement etc.). The procedure is versatile and can be applied to a variety of neural recording modalities including single, multi-unit, and continuous field potential recordings. Furthermore, because the algorithm estimates the transfer functions between every stimulation and neural recording electrode, the procedure can be applied irrespective of the stimulation currents used. It is thus compatible with single and multi-site stimulation, high-rate stimulation, and is applicable to electrical stimuli with arbitrary pulse amplitudes and shapes. By applying the procedure to sample neural datasets (single and multi-channel stimulation), we demonstrate a vast signal-to-noise ratio improvement of ∼25−40 dB.

## Materials and Methods

### Artifact Prediction and Removal

#### Multi-Input Multi-Output Artifact Prediction Wiener Filter

We develop an optimal Wiener filter algorithm to predict neural recording artifacts upon delivering electrical stimulation currents on a multi-channel stimulating electrode array. The predicted artifacts are then subtracted from the actual neural recording trace to yield a noise reduced estimate of the neural activity.

We assume a generalized multi-input (stimulation) multi-output (recording) framework for developing a linear filter approximation of the recording artifact. Given that electrical stimulation artifacts are the result of linear capacitive and inductive coupling between the stimulating and recording electrodes (Rivnay et al., 2017), we model the transformations between the electrical stimulus and recorded artifact as a linear Wiener filter with unknown impulse response (or equivalently, transfer function). Each stimulating and neural recording electrode pair has its own characteristic transfer function and thus a unique impulse response, which can be determined based on the input and output data. The composite multi-site stimulation artifact is modeled as a linear sum of the artifacts generated by each stimulation channel and thus we have:

(1) $$y_{m}\left[k\right]=\sum_{n=1}^{N}x_{n}{\left[k\right]}^{*}h_{nm}\left[k\right]\;m=1,\ldots ,M$$

where *k* is the discrete time index, ^∗^ is the discrete convolution operator, *y*_*m*_[*k*] is the predicted artifact for channel *m* (**y**_*m*_ in vector form), *h*_*nm*_[*k*] is the impulse response between the *n*-th stimulation channel and *m*-th neural recording channel (**h**_*nm*_ in vector form), and *x*_*n*_[*k*] is the electrical stimulation signal applied to stimulation channel *n* (**x**_*n*_ in vector form). In matrix form **y** = **hx** where **y** = [**y**_1_⋯**y**_*M*_] is a matrix containing the predicted outputs for the *M* recording channels, **x** = [**x**_1_⋯**x**_*N*_] is a matrix containing the input electrical stimulation signals across *N* stimulation channels, and:

(2) $$\text{h}=\left[\begin{array}{ccc} {\text{h}}_{11} & \ldots & {\text{h}}_{1M}\\ \vdots & \ddots & \vdots \\ {\text{h}}_{N1} & \ldots & {\text{h}}_{NM} \end{array}\right]$$

is an *N*x*M* matrix containing the impulse response vectors (**h**_nm_) between all stimulation and recording channels. The impulse responses are represented as column vectors, **h**_*nm*_ = [*h*_*nm*_[0] ⋯ *h*_*nm*_[*L* − 1]]^*T*^, which contain the impulse response time coefficients between the *n*-th input and *m*-th output, where *L* represents the filter order. Since there are a total of *L* samples for each of the impulse response vectors, the matrix **h** contains a total of *NLxM* coefficients.

The goal is to derive the filter matrix **h** using experimental measurements. The estimated filter matrix can then be used to predict the recorded artifacts. The optimal solution that minimizes the mean squared error of the predicted artifact is obtained via the Wiener-Hopf equation (Hayes, 1996).

(3) $$\hat{\text{h}}={\left({\text{C}}_{\mathrm{xx}}\right)}^{-1}{\text{R}}_{\mathrm{yx}}$$

where $\hat{\text{h}}$ is the filter matrix solution that minimizes the mean squared error between the predicted and real artifacts,

(4) $${\text{C}}_{\mathrm{xx}}=\left[\begin{array}{ccc} {\text{c}}_{x_{1}x_{1}} & \ldots & {\text{c}}_{x_{1}x_{N}}\\ \vdots & \ddots & \vdots \\ {\text{c}}_{x_{N}x_{1}} & \ldots & {\text{c}}_{x_{N}x_{N}} \end{array}\right]$$

represents the stimulation signal covariance matrix which contains correlation functions (**c**_**x**_*n*_**x**_*l*__) between the *n*-th and *l*-th (*l*,*n* = 1,…,*N*) input channels, and:

(5) $${\text{R}}_{\mathrm{yx}}=\left[\begin{array}{ccc} {\text{r}}_{y_{1}x_{1}} & \ldots & {\text{r}}_{y_{1}x_{N}}.\\ \vdots & \ddots & \vdots \\ {\text{r}}_{y_{M}x_{1}} & \ldots & {\text{r}}_{y_{M}x_{N}}. \end{array}\right]$$

is a matrix containing the cross-correlation functions between the *m*-th output and *n*-th input channels (**r**_**y**_*m*_**x**_*n*__).

Upon deriving the multi-site filters using Eqn. 3, $\hat{\text{h}}$, the stimuli artifacts are then predicted by convolving each of the estimated sub-filter impulse responses, ${\hat{\text{h}}}_{nm}$, with the corresponding input signals and applying Eqn. 1. Finally, the predicted artifacts are subtracted from the recorded data yielding the noise-reduced estimate of the neural traces. Although Eqn. 3 is derived for multi-input multi-output (*N* > 1, *M* > 1) neural recording and stimulation scenarios, the procedure is also compatible with multi-input single-output (*N* > 1, *M = 1*), single-input multi-output (*N = 1*, *M* > 1) and single-input single-output (*N = 1*, *M = 1*) neural stimulation and recording scenarios.

As a note, we point out that the form of the *predictive* Wiener filter used here differs from *blind deconvolutional* Wiener filters used in previous reports by others for artifact removal which assume that the artifact-generating signals are unknown (Izzetoglu et al., 2005; Zhi and Chin, 2006; Sweeney et al., 2012). Deconvolutional filters use the signal and noise spectrum statistics to optimally reject the artifact signal via deconvolution. In general, because the signal and noise spectrums often overlap, such approaches tend to distort the neural signals of interest upon removing the artifacts and are not intended to fully remove the artifact. In our case, the Wiener filter is instead used to predict the recorded artifact from *known* inputs, which can then be removed from the neural recording by subtraction without distorting the neural signal.

### Linearity Assessment and Artifact Removal Quality

#### Linearity Assessment

The principal underlying assumption required for the proposed artifact prediction and removal method is the linear relationship between the stimulation current and recorded artifact. Such a relationship is expected given the passive conduction properties of the tissue and the capacitive or inductive coupling with the stimulation current at the recording electrode interface (Trebaul et al., 2016; Rivnay et al., 2017). However, it has been suggested that recording artifacts can behave in a non-linear fashion (Mena et al., 2017), which would limit the general applicability of the proposed approach. Thus, we quantified the extent of the linearity (or non-linearity) of the stimulus current-artifact relationship by explicitly testing the *scaling* and *additivity* properties, which are requisites for a linear system (Lathi, 2000). First, for each of the recording scenarios tested, we delivered currents of varying amplitudes. This allowed us to explicitly test how artifact amplitudes *scale* with respect to the input current amplitudes. We also performed a set of experiments in which we concurrently delivered current pulses across multiple electrodes (see section below: “Recording in the Rat Auditory Midbrain and Cortex”). This second set of recordings allowed to test how multiple stimulus currents *add* together to generate a composite artifact.

#### Signal to Noise Ratio Estimation

We used a shuffled trial procedure to *estimate* the artifact (noise) and neural signal power spectra which were then used to estimate the signal-to-noise ratio (SNR) of the neural recording or the artifact reduction ratio (ARR). The procedure requires that we deliver an identical electrical stimulation signal from two trials in order to estimate the signal and noise power spectrum. Consider a recorded neural trace:

(6) $$\text{y}={\text{y}}_{n}+{\text{y}}_{a}$$

where y_*n*_ represents the artifact-free neural trace (i.e., no artifact) and y_*a*_ represents the recorded artifact. If y′ represents the data recorded in the second trial of a repeated experiment (i.e., same electrical stimulation signal) then the artifact should be identical between the two trials (y_*a*_) so that:

(7) $${\text{y}}^{\prime }={\text{y}}_{n}^{\prime }+{\text{y}}_{a}$$

where here, ${\text{y}}_{n}^{\prime }$, is the neural response component for the second trial. This component differs from the first trial response (y_*n*_) because of neural variability. Computing the cross-spectral density (CSD) between the two trials yields:

(8) $${\text{Φ}}_{{\text{yy}}^{\prime }}\left(\omega \right)={\text{Φ}}_{{\text{y}}_{n}{\text{y}}_{n}^{\prime }}\left(\omega \right)+{\text{Φ}}_{{\text{y}}_{n}{\text{y}}_{a}}\left(\omega \right)+{\text{Φ}}_{{\text{y}}_{a}{\text{y}}_{n}^{\prime }}\left(\omega \right)+{\text{Φ}}_{{\text{y}}_{a}{\text{y}}_{a}}\left(\omega \right)$$

Similarly, the power spectral density (PSD) of the first trial is:

(9) $${\text{Φ}}_{\text{yy}}\left(\omega \right)={\text{Φ}}_{{\text{y}}_{n}{\text{y}}_{n}}\left(\omega \right)+{\text{Φ}}_{{\text{y}}_{n}{\text{y}}_{a}}\left(\omega \right)+{\text{Φ}}_{{\text{y}}_{a}{\text{y}}_{n}}\left(\omega \right)+{\text{Φ}}_{{\text{y}}_{a}{\text{y}}_{a}}\left(\omega \right)$$

**FIGURE 1:**
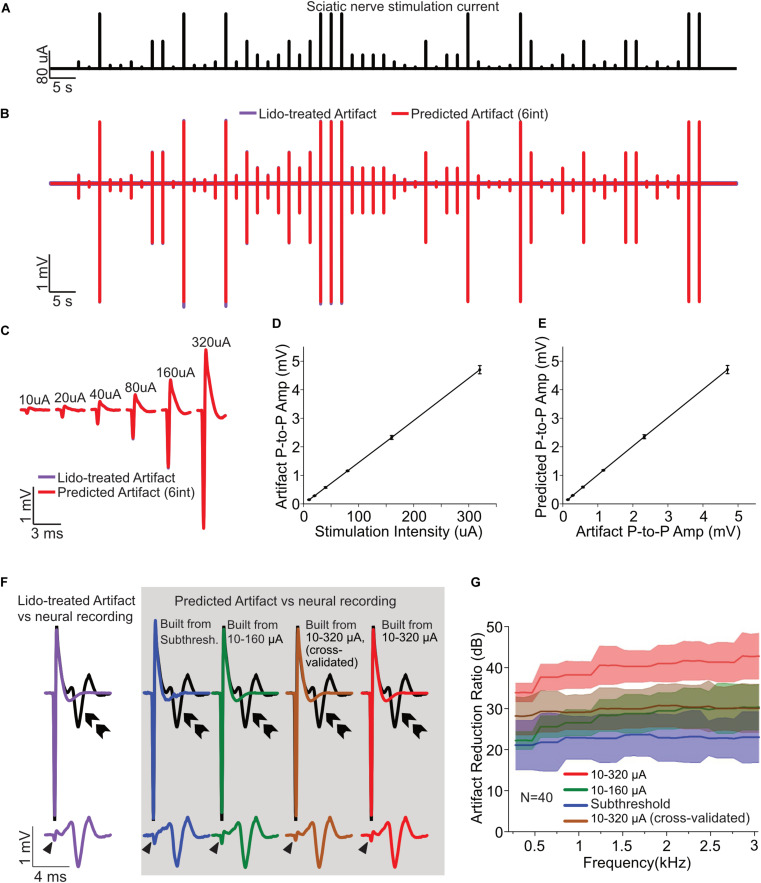
Artifact removal from neural recordings in mouse sciatic nerve. **(A)** Electrical stimulation current signal (120-s duration, 0.5 Hz, 0.2 ms duration, cathodic current) with six amplitudes (10, 20, 40, 80, 160, and 320 μA; 10 stimuli per amplitude condition) delivered in pseudo random order. **(B)** Experimentally recorded artifacts after lidocaine treatment (purple) overlapped with the Wiener filter-predicted artifact (red). **(C)** Magnified views of the recorded artifacts (lidocaine, purple) superimposed with predicted artifacts (Wiener filter, red) from different stimulating amplitudes. **(D)** Input current amplitudes and the peak-to-peak amplitudes of the recorded post-lidocaine artifacts follow a linear relationship (r^2^ = 0.9997 ± 0.0004, Mean ± SD, *N* = 40). **(E)** The peak-to-peak amplitudes of the recorded (lidocaine) and predicted (Wiener filter) artifacts follow a linear relationship (r^2^ = 0.9997 ± 0.0004, Mean ± SD, *N* = 40). **(F)** The suprathreshold recordings (pre-lidocaine treatment, 320 μA; black curves) are superimposed with the post-lidocaine artifact (purple, left). The predicted (Wiener filter) artifacts of four estimation scenarios (colored, in gray box, top) are shown along with the isolated action potentials after artifact removal (gray box, bottom). Purple: lidocaine treated artifact (top); Blue: predicted artifact using the strongest subthreshold current estimation (10 trials); Green: predicted artifact using the lowest five current estimation (10–160 μA, 50 trials); Brown: predicted artifact using the lowest five current (10–160 μA) along with 5 trials of 320 μA current estimation (55 trials). Red: predicted artifact using all the six-current estimation (10–320 μA, 60 trials). The isolated action potentials (obtained by subtracting the predicted artifacts from suprathreshold responses) are displayed in the bottom row (same color scheme). Arrow head indicates the artifact residue after the subtraction; double arrow indicates nerve activity evoked from the fiber. **(G)** The artifact reduction ratio for the four estimation scenarios are shown with shaded error bar (Mean ± SD; *N* = 40 fibers). The ARR achieved using the highest subthreshold current estimation method is 22.8 ± 4.4 dB (blue), using the lowest five current estimation method is 28.1 ± 3.5 dB (green), using the lowest five current along with 5 trials of 320 μA current estimation method is 29.9 ± 4.7 dB (brown), using all the six-current estimation is 39.9 ± 3.3 dB (red). The average ARR are calculated within 300–3000 Hz.

**FIGURE 2:**
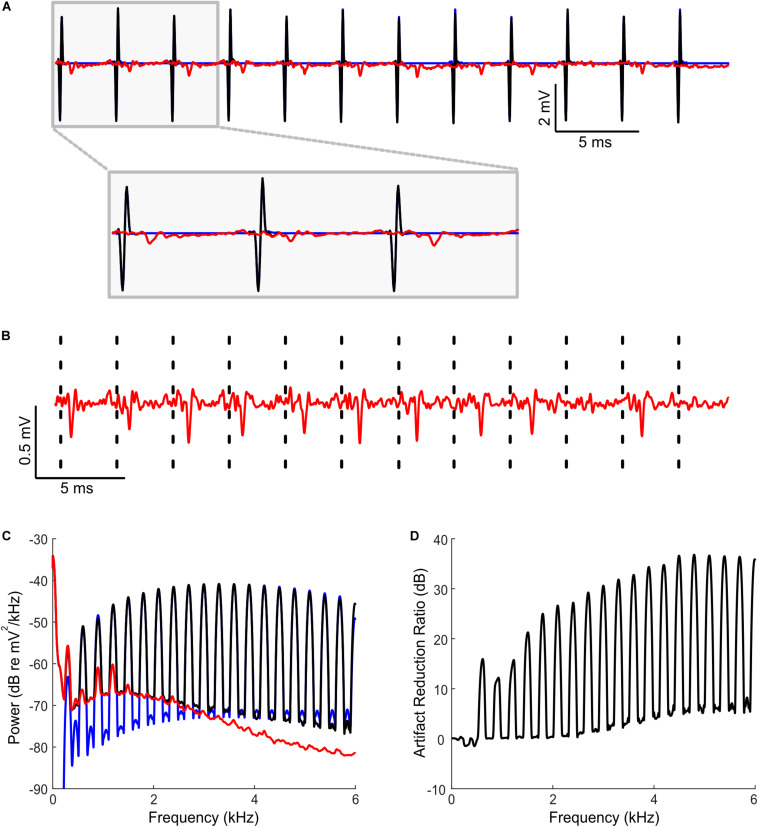
Artifact removal during bilateral cochlear implant stimulation and concurrent extracellular recordings in rat inferior colliculus. **(A)** Sequences of binaural biphasic pulses were delivered at 300 Hz stimulation rate (0 ms interaural time delay shown; 10 sequences delivered; each sequence lasting 200 ms duration; the segment shown is between 10 and 50 ms post onset). Example segment containing a raw neural recordings (black) and predicted artifacts (blue) demonstrates that both are highly overlapped. The cleaned neural recording trace obtained by subtracting the predicted artifact from the original recording (red, superimposed) show no visible signs of artifact signals. **(B)** Zoomed version of the cleaned neural recording signal (red). Dashed lines indicate the time instances of the recorded artifacts. **(C)** Power spectrum of the neural recording before (black) and after (red) artifact removal. The artifact spectrum contains energy at harmonics of the 300 Hz fundamental frequency of the stimulus. The predicted artifact spectrum (obtained as the cross spectrum between recording trials, see “Materials and Methods”) is shown in blue and largely overlaps the recorded spectrum prior to artifact removal (black). **(D)** Shows the artifact reduction ratio. Artifacts are reduced by an average of 27.2 dB (measured at harmonics of 300 Hz).

**FIGURE 3:**
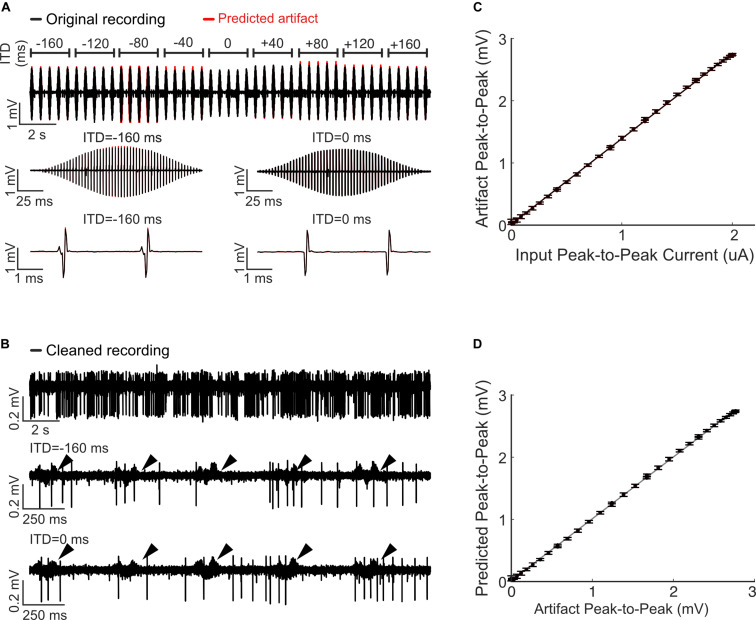
Predicting and removing time-varying artifacts and testing for linearity during bilateral cochlear implant stimulation. **(A)** Neural recordings were obtained at multiple interaural time differences (–160 to +160 us; 40 us steps) using 300 Hz pulse trains modulated with a Hanning window (see “Materials and Methods”). The predicted artifacts (red) are shown at multiple magnifications and closely match the recorded artifacts (black). **(B)** Cleaned recordings obtained by subtracting the Wiener filter predicted artifacts from the original recordings are shown at two different time scales (ITD = −160 ms, middle; ITD = 0 ms, bottom). **(C)** The input current peak-to-peak amplitude and the recorded peak-to-peak voltage of the artifacts exhibit an exceptionally high correlation (r^2^ = 0.9981± 0.0001; Mean ± SEM), indicative of a linear relationship. **(D)** The predicted and recorded artifact peak-to-peak amplitudes are likewise highly correlated and consistent with a linear input-output relationship. The data shown in panels **(C,D)** are for the 0 ms ITD condition. Error bars designate SD.

**FIGURE 4:**
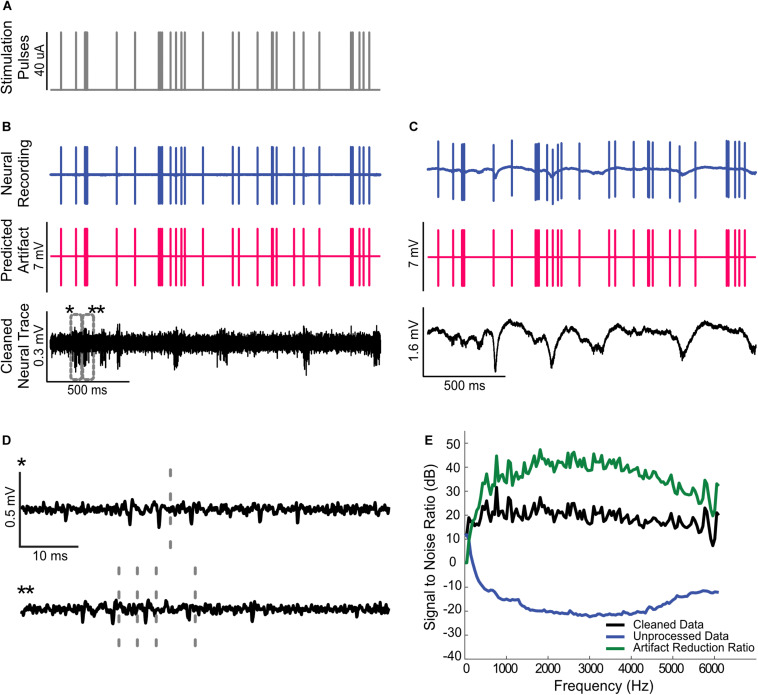
Artifact removal during a single channel electrical stimulation of the auditory midbrain and concurrent recording in auditory cortex. **(A)** Random Poisson distributed pulse sequence (average pulse rate of 16 Hz) were delivered to an electrode in auditory midbrain of a rat. Highpass filtered **(B)** and raw **(C)** neural recordings from a cortical electrode are dominated by the electrical artifacts (top). The estimated Wiener filters are used to predict the recorded artifacts (middle panels). Subtracting the artifacts from the neural recordings yields noise reduced estimates of the neural activity (bottom). **(D)** Zoomed sample waveforms showing the filtered extracellular signals after artifact subtraction (* and ** from panel **(B)**, bottom). **(E)** Signal to noise ratio prior to and after subtraction of the predicted artifacts (blue and black curve, respectively). The artifact reduction ratio is superimposed on the same panel (green curve).

Given that the artifact signal is reproducible across trials and typically much larger than the recorded neural activity (e.g., as seen for the examples of Figures 1–4), the artifact term in Eqn. 8 dominates:

(10) $${\text{Φ}}_{{\text{yy}}^{\prime }}\left(\omega \right)\approx {\text{Φ}}_{{\text{y}}_{a}{\text{y}}_{a}}\left(\omega \right)$$

so that CSD between trials approximates the artifact noise spectrum. Furthermore, we note that for sufficiently long recordings, ${\text{Φ}}_{{\text{y}}_{a}{\text{y}}_{n}}\left(\omega \right)and{\text{Φ}}_{{\text{y}}_{a}{\text{y}}_{n}^{\prime }}$ (ω) yield identical spectrum estimates on average and that ${\text{Φ}}_{{\text{y}}_{n}{\text{y}}_{n}}\left(\omega \right)\gg {\text{Φ}}_{{\text{y}}_{n}{\text{y}}_{n}^{\prime }}$ (ω) as a result of neural trial variability between trials. Thus, the neural signal spectrum can be approximated by subtracting the CSD from the PSD.

(11) $${\text{Φ}}_{{\text{y}}_{n}{\text{y}}_{n}}\left(\omega \right)\approx {\text{Φ}}_{\text{yy}}\left(\omega \right)-{\text{Φ}}_{{\text{yy}}^{\prime }}\left(\omega \right)$$

The signal to noise ratio is then approximated by:

(12) $$\text{SNR}\left(\omega \right)=\frac{{\text{Φ}}_{Signal}\left(\omega \right)}{{\text{Φ}}_{Noise}\left(\omega \right)}\approx \frac{{\text{Φ}}_{\text{yy}}\left(\omega \right)-{\text{Φ}}_{{\text{yy}}^{\prime }}\left(\omega \right)}{{\text{Φ}}_{{\text{yy}}^{\prime }}\left(\omega \right)}$$

In the above, all cross and power spectral density estimates were obtained using a Welch average periodogram and a Kaiser window (β = 5, *N* = 256 time samples or 21 ms). To confirm the validity of the approximations used to derive Eqn. 12, we also estimated the SNR using an artifact free neural recording segment. Φ_*Signal*_(ω) was estimated by collecting a 15-second neural trace without any electrical stimulation, which we then used to estimate the signal spectrum. We also estimated the noise spectrum directly from the Wiener filter predicted artifacts by computing the spectrum of the predicted artifact. Both procedures produce quantitatively similar results when compared to the original estimates (within < 3 dB) confirming the validity of the approximations used to derive Eqn. 12.

#### Artifact Reduction Ratio (ARR)

In addition to defining the SNR, we also defined and measured an artifact reduction ratio (ARR). This metric quantifies the reduction in artifact power following artifact removal and thus provides a measure of the artifact removal quality. It is defined as:

(13) $$\text{ARR}\left(\omega \right)=\frac{{\text{SNR}}_{post}\left(\omega \right)}{{\text{SNR}}_{pre}\left(\omega \right)}=\frac{{\text{Φ}}_{Noise,pre}\left(\omega \right)}{{\text{Φ}}_{Noise,post}\left(\omega \right)}$$

where SNR_*pre*_(ω) is the SNR prior to artifact removal and SNR_*post*_(ω) is the measured SNR after applying the artifact removal algorithm. Since the neural signal spectrum is unchanged by the artifact removal procedure, the above can also be estimated directly by taking the ratio of the noise spectrum prior to (Φ_*Noise*,*pre*_(ω)) and post-removal of the artifact Φ_*Noise*,*post*_(ω)). For the aperiodic stimulation used in inferior colliculus (described below), we note that the ARR metric is well defined for all frequencies since, in that case, the signal and noise spectrum is continuous at all frequencies. However, for periodic electrical stimulation such as in the cochlear implant study (e.g., electrical stimulation periodically at 300 Hz, described below), the electrical stimulation produced periodic artifacts with harmonic components in the signal spectrum at multiples of the stimulation frequency. Thus, the signal spectrum and hence the ARR contains signal components only at harmonics of the stimulation frequency and are thus well defined only at these components.

Depending on the data that were available, the ARR was estimated in one of two ways. For the sciatic nerve recordings (see section below: “Mouse Sciatic Nerve Recordings”), the isolated artifacts were obtained during the treatment of lidocaine, which is a non-selective sodium channel blocker that blocks virtually all neural activities in the sciatic nerve. Thus, for this condition, there was no need to remove the neural signal spectrum numerically in order to isolate the artifact spectrum prior to estimating the ARR (as in Eqn. 11). The spectrum prior to artifact removal was obtained as the spectrum of the original lidocaine recording (pre-artifact removal), while the spectrum post-artifact removal was obtained by subtracting the predicted artifact from the original lidocaine recording (using the Wiener filter method) and subsequently computing the power spectral density. For both, the cochlear implant stimulation and auditory midbrain stimulation recordings, neural activity and artifacts were not isolated chemically using lidocaine. Thus, we estimated the artifact and neural spectrums and the corresponding ARR numerically using shuffled cross-spectral density procedure as described above (Eqns. 6−12).

### Mouse Sciatic Nerve Recordings

#### Surgical Procedures

All procedures were approved by the University of Connecticut Institutional Animal Care and Use Committee. Sciatic nerves of male C57BL/6 mice (6–8 weeks, Taconic, Germantown, NJ, United States) were harvested for extracellular recordings from teased nerve filaments as detailed previously (Chen et al., 2017; Ilham et al., 2018). Mice were anesthetized by isoflurane inhalation, euthanized by exsanguination from perforating the right atrium, and perfused through the left ventricle with oxygenated Krebs solution (in mM: 117.9 NaCl, 4.7 KCl, 25 NaHCO_3_, 1.3 NaH_2_PO_4_, 1.2 MgSO_4_, 2.5 CaCl_2_, and 11.1 D-glucose). Bilateral sciatic nerves of ∼30 mm long were harvested from their proximal projection to the L4 spinal cord to their distal branches innervating gastrocnemius muscles and transferred to a custom-built chamber perfused with oxygenated Krebs solution at 30°C. The distal end of the sciatic nerve (∼5 mm) was gently pulled into a recording compartment filled with mineral oil and carefully split (i.e., teased) into fine neural filaments (∼25 μm thick) for extracellular recordings of action potentials.

#### Stimulation and Recording in the Sciatic Nerve Preparation

Action potentials were evoked at the un-teased end of the sciatic nerve using a platinum-iridium electrode (FHC Inc., ME, United States). Electrical currents were delivered using a sub- and supra-threshold stimulation protocol consisting of a 120-s long low-frequency stimulations (0.5 Hz, 0.2 ms duration, cathodal current) with six amplitudes delivered in pseudo random order (10, 20, 40, 80, 160, and 320 μA; 10 stimulus pulses per amplitude condition) as shown in Figure 1A.

Extracellular recordings from multiple teased nerve filaments were conducted by a custom-built 5-channel electrode array consisting of micro-wires deployed parallel to each other with ∼150 μm clearance as described previously (Chen et al., 2017; Ilham et al., 2018). Recordings were digitized at 25 kHz, band-pass filtered (300−3000 Hz) and stored on a PC using an integrated neural recording and stimulating system (IZ2H stimulator, PZ5-32 neurodigitizer and RZ5D processor, TDT, Alchua, Florida, United States).

#### Application of Lidocaine for Acquiring Isolated Artifacts

To quantify the efficiency of artifact removal via the Wiener filter artifact removal method, we used a non-selective sodium channel blocker (lidocaine) to remove most if not all neural activity, which allowed us to obtain recordings of isolated artifacts. A bronze tube (4 × 4 mm cross section) was placed over the sciatic nerve to isolate a small segment of the nerve trunk (∼4 mm) for lidocaine application. On both edges are small notch holes to allow nerve trunk to go through, which were lined with petrolatum to prevent solution exchange between inside and outside the bronze tube. Krebs solution inside the bronze tube was replaced with lidocaine (2% dissolved in saline, ∼0.2 ml) for 5 min, and then the bronze tube was removed for bath washout. The same stimulation protocol mentioned above was conducted immediately after lidocaine application to obtain isolated artifacts for the six-amplitude stimulation current signal.

The benefits of applying lidocaine are as follows. First, lidocaine treatment prevents action potential generation, which allows us to isolate the artifact signal in the absence of neural activity. This is useful for validating the accuracy of the artifact prediction since there is no confounding neural activity. Second, the prediction filters obtained during lidocaine treatment were also used to predict and remove the artifacts obtained in the absence of lidocaine treatment during supra-threshold stimulation. Thus, the approach allows us to cross validate our artifact removal algorithm by comparing the Wiener filter artifact cancelation performance against the pure artifact recordings under lidocaine treatment at supra-threshold stimulation levels.

#### Estimating Artifact Prediction Filters and ARR

Artifact prediction filters were estimated using Eqn. 3 for four different scenarios. First, we used the highest subthreshold current (without evoking action potentials) to estimate the artifact prediction filters using Eqn. 3. For this condition, there were only 10 pulses delivered so that the artifact prediction filter was estimated using only 10 measurements. We refer to this condition as the subthreshold filter. This subthreshold method of estimating the Wiener filter was only used for the sciatic nerve recordings and was not used subsequently for the cochlear implant or auditory midbrain stimulation. Next, we used lidocaine treated recordings, which lack neural activity, to derive the artifact prediction filters using three approaches. For the first lidocaine condition, we used the recordings containing currents between 10 and 160 μA to derive the Wiener filter using Eqn. 3. Next, we used the recordings containing stimulus currents between 10 and 160 μA along with the first five trials of 320 μA artifacts to estimate the Wiener filters and then performed cross validation by comparing the predicted artifacts with those from the remaining five trials at 320 μA stimulation. Finally, we estimated the Wiener filers using all of the recorded data from both sub- and supra-threshold stimulation under lidocaine treatment (10−320 μA).

These four filters were then used to predict the stimulation artifacts during the 320 μA current stimulation scenario, which were subtracted from the neurophysiological recordings to isolate the supra-threshold nerve response evoked by 320 μA current stimulation. The artifact removal quality was assessed with the ARR defined above (Eqn. 13) for each scenario.

### Bilateral Cochlear Implant Stimulation in Rats

#### Surgical Procedures

To illustrate the artifact removal during CI stimulation, we used example data from two female Wistar rats, one of which was normally hearing, the other neonatally deafened by daily intraperitoneal (i.p.) injections of 400 mg/kg kanamycin from postnatal day 9 to 20 (Osako et al., 1979; Rosskothen-Kuhl and Illing, 2012). The animals were part of studies designed to determine factors governing sensitivity to binaural cues delivered via direct, intracochlear stimulation similar to that used in clinical CI devices. These data were obtained at the City University of Hong Kong, using procedures licensed by the Department of Health of Hong Kong (license number 16−52 DH/HA&P/8/2/5) and approved by the Animal Research Ethics Subcommittee of City University. All surgical procedures, including CI implantation and craniotomy, were performed under anesthesia, which was induced with an i.p., injection of ketamine (80 mg/kg) and xylazine (12 mg/kg) and maintained by continuous i.p., infusion of ketamine (17.8 mg/kg/h) and xylazine (2.7 mg/kg/h) in 0.9% saline solution at a rate of 3.1 ml/h, and the animal’s body temperature was maintained at 38°C using a feedback-controlled heating pad (RWD Life Sciences, Shenzhen, China). The cochlear implantation methods are described in detail in Rosskothen-Kuhl and Illing (2012); Rosskothen-Kuhl et al. (2018).

In short, four rings of an eight-channel intracochlear electrode carrier (ST08.45, Peira, Beerse, Belgium) were inserted through a cochleostomy in the medio-dorsal direction into the middle turn of both cochleae. The tip electrode ring of each intracochlear array was used to deliver electrical stimuli, while the second, adjacent electrode served as ground. A craniotomy was then performed bilaterally of the central cranial suture, just anterior to lambda, and a single-shaft, 32-channel silicon array electrode (ATLAS Neuroengineering, E32-50-S1-L6) was inserted stereotaxically into the inferior colliculus (IC) through the overlying occipital cortex using a micromanipulator (RWD Life Sciences).

#### Electrophysiology

Electrical stimuli were generated using a Tucker Davis Technology (TDT, Alachua, Florida, United States) IZ2MH programmable constant current stimulator (TDT, Alachua, Florida, United States) running at a sample rate of 24414 Hz. To verify that the cochlear implantation was successful and yielded symmetric evoked responses at comparatively low thresholds (typically less than 100 μA peak) in each ear, electrically evoked auditory brain stem response thresholds were measured for each ear individually. This was done by recording scalp potentials with subcutaneous needle electrodes implanted over the vertex and each bulla, averaged over the presentation of 400 individual biphasic electrical stimulus pulses.

Extracellular signals were recorded at a rate of 24414 Hz with a TDT RZ2 with a NeuroDigitizer headstage and BrainWare software. Neural tuning to interaural time differences (ITDs) of binaurally delivered pulse trains was then measured by recording extracellular responses of IC neurons to 200 ms long trains of anode leading, biphasic electrical pulses (duty cycle: 40.96 μs positive, 40.96 μs at zero, and 40.96 μs negative), with peak pulse amplitudes approximately 6 dB above neural response thresholds and a rate of 300 pulses per second. The pulses were delivered bilaterally to both ears and the ITD between the left and right ear was varied (ITD = −160, −80, −40, 0, +40, +80, and +160 μs). In one set of recordings, the amplitude of the pulse sequences was modulated with a Hanning window. This allowed us to test for linearity of the stimulus current to artifact relationship and ultimately allows us to determine whether the Wiener filter artifact prediction method is able to generalize and predict dynamic time-varying artifacts. The recordings typically exhibited short response latencies (≈ 3−5 ms), which indicates that they probably come predominantly from the central region of IC.

Using the suprathreshold recording traces, we applied Eqn. 3 and derived the Wiener artifact prediction filters for each recording. The estimated filters were then used to predict and subsequently subtract the recording artifacts from the recorded traces. Eqn. 3 was applied in a variety of ways in order to demonstrate the flexibility of the Wiener filter method. For the first recording, we treated each ITD condition separately, and derived one artifact prediction filter per condition (Figure 3, shown for 0 ms ITD). For this example, the artifacts associated with each ITD condition are highly reproducible because the pulse amplitude and ITD of the left and right ear pulse was not varied for each individual condition. This allows us to measure a single composite artifact filter for each individual ITD condition. For the second example (Figure 3), we used pulses that varied dynamically over time and we included all ITD conditions during the filter estimation. For this example, each ITD produces a unique artifact waveforms and the goal was to derive filters that could generalize across all of the recorded conditions. We did so by treating the pulse sequences of the left and right ear as distinct inputs (2 input Wiener filter). Thus, for each recording channel, we obtained two separate filters, one for the left and the other for the right channel. These filters were then individually convolved with the left and right ear pulse sequence (with the appropriate ITD) and subsequently summed to derive the final predicted artifact (Eqn. 1 for *N* = 2). Finally, we tested the quality of the artifact prediction achieved either by applying Eqn. 3 one recording channel at a time or by considering all recording channels simultaneously (in matrix form, multi-output scenario). Regardless of which approach we used to estimate the artifact prediction filters, the results were identical and within the machine precision (e.g., RMS error for example of Figure 3 is 1.4× 10^−29^ %).

### Electrical Stimulation and Recording in the Rat Auditory Midbrain and Cortex

#### Surgical Procedures

All procedures were approved by the Institutional Animal Care and Use Committee of the University of Connecticut. Recordings were obtained from right cerebral hemisphere of adult male Brown Norway rats. Anesthesia was induced with ketamine and xylazine and maintained throughout the surgery and recording procedures. Depth of anesthesia was monitored using pedal reflex, heart rate, and blood oxygen saturation (SpO2) measured by a pulse oximeter. A heating pad was also used to maintain the animal’s body temperature at 37.0 ± 1.0°C. Craniotomies were performed over the temporal cortex to make both cortex and IC regions accessible. Dexamethasone and atropine sulfate were administered to reduce cerebral edema and secretions in the airway.

#### Electrophysiology

Sixteen channel acute neural recording probes (NeuroNexus 5 mm probe; 16-linear spaced sites with 150 um separation; site impedance ∼100 KΩ) were used to record neural activity and also to deliver electrical stimulation to the IC. Stimulating and recording probes were grounded to the animal’s neck muscle and the eye bars holding the animal in place, respectively (Lim and Anderson, 2006). The probes were inserted with a high precision LS6000 microdrive (Burleigh EXFO). A 4-channel acute single-shank recording tetrode (Qtrode, NeuroNexus Inc; 5 mm shank length, tetrode with 25 um site separation; site impedance ∼1−3 MΩ) was simultaneously inserted into auditory cortex (AC). Penetration sites were chosen within the depth range of cortical layer IV where AC receives its inputs from auditory thalamus. A sequence of pure tones with varying frequency and attenuation was initially played to the animal’s left ear (contralateral to the brain opening) and brain responses were recorded to generate frequency response areas (FRA) to verify probes placements in the central nucleus of IC and AC.

**FIGURE 5:**
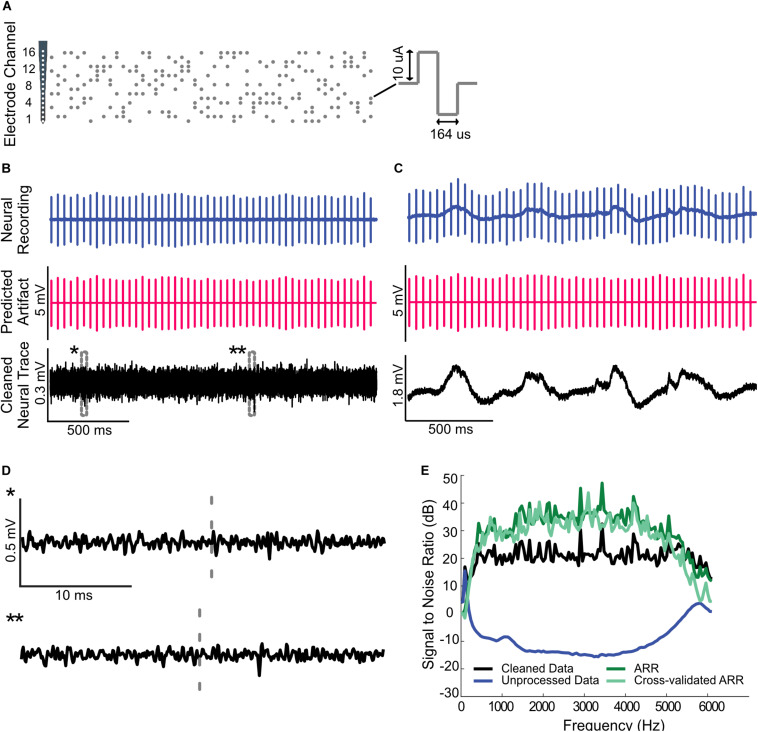
Artifact removal during high throughput multi-site electrical stimulation. **(A)** Spatio-temporal pulse sequence applied to a 16-channel probe placed in the auditory midbrain of a rat. Highpass filtered **(B)** and raw **(C)** neural recordings from a cortical electrode are dominated by the electrical artifacts (top). The estimated multi-channel Wiener filters are used to predict the recorded artifacts (middle panels). Subtracting the artifacts from the neural recordings yields noise reduced estimates of the neural activity (bottom). **(D)** Zoomed sample waveforms showing the filtered extracellular signals after artifact subtraction (* and ** from panel **(B)**, bottom). **(E)** Signal to noise ratio prior to and after subtraction of the predicted artifact is superimposed on the same panel (gray and black curve, respectively). The artifact reduction ratio obtained using the whole data segment and the cross-validated ARR obtained using half of the data are superimposed on the same panel (dark and light green curves, respectively).

**FIGURE 6:**
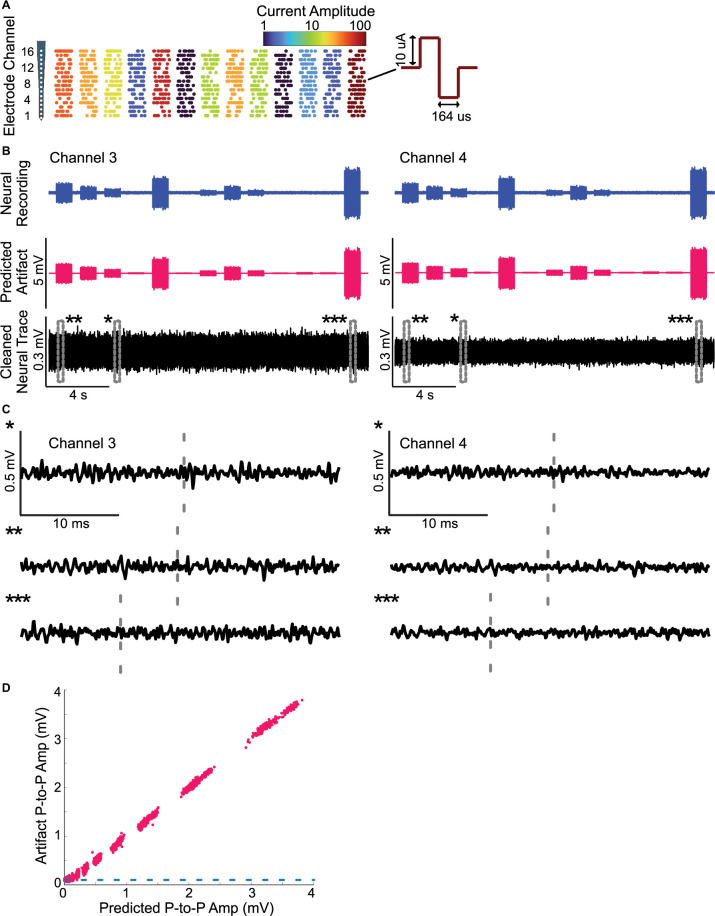
Removing artifacts during dynamic multi-site electrical stimulation and testing for linearity. **(A)** Dynamically time-varying current pulse sequences were delivered across 16-channel recording probe in the rat auditory midbrain. Stimulation sequences consist of concurrent activated channels (4 randomly selected channels; every 40 ms) with pulse amplitudes that vary dynamically and randomly (between 0.1 and 10 μA, color indicates current strength; log-steps; see section “Materials and Methods”). **(B)** The recorded neural traces and predicted artifacts are shown for two of four recording channels along with the cleaned neural traces. The multi-channel Wiener filter accurately predicts the recorded artifacts and there no evident signs of residual artifacts upon removal **(C)** The magnified cleaned neural traces (*, **, and *** from panel **(B)**, bottom) shown no visible artifact distortions. The gray dashed lines indicate time instants that contained visible artifacts before removal. **(D)** The predicted and actual recorded artifact peak-to-peak amplitudes exhibit a high correlation (r^2^ = 0.9981± 0.0001, Mean ± SEM) suggesting that the artifacts are linearly related to the input currents.

Neural activity was recorded digitally at a sampling rate of 12 kHz using a PZ2 preamplifier and RZ2 real time processor (TDT, Alchua, Florida, United States). Electrical stimuli were delivered to the IC electrode via the IZ2 stimulation module (TDT, Alchua, Florida, United States). Electrical pulse sequences with amplitudes of either 40 or 10 μA were transmitted to a single electrode (Figure 4) or independently across multiple electrode channels (Figures 5, 6), respectively (see below for details). Neural activity was then recorded from the auditory cortical probe for the duration of each stimulus.

#### Single-Channel and Multi-Channel Electrical Stimulation

We first delivered Poisson-distributed biphasic pulse sequence during single channel electrical stimulation. A random sparse sequence of impulses with arrival time following Poisson point process and impulse rate of 16 Hz was first generated (86 s duration; delivered twice). The impulse sequence was convolved with a biphasic pulse (164 μs duration and 40 μA current amplitude) to produce the current waveform used for electrical stimulation.

For multi-site electrical stimulation, we delivered a random quad-pulse train sequence (RQP; 86 s duration; delivered twice). The RQP sequence is generated by delivering biphasic pulses (164 μs duration and 10 μA amplitude) concurrently across 4 randomly chosen electrode channels every 40 ms yielding an average pulse rate of 100 pulses/s as illustrated in Figure 5. This multi-site sequence produces a random spatio-temporal patterned set of pulses that are delivered across the 16-channel electrode array. We also delivered an RQP sequence in which the amplitude of the pulses was varied dynamically over time (Figure 6). Pulse amplitudes for this sequence varied between 0.1 and 10 μA in logarithmic steps (11 steps total). Because the pulse amplitudes scale over two orders of magnitude and the pulses summate across channels, this multi-channel sequence allows us to test for linearity of the current-artifact relationship.

## Results

We demonstrate the Wiener filter effectiveness for predicting and removing neural recording artifacts during single and multi-channel electrical stimulation for both high-frequency spiking activity and low-frequency local field potentials (LFP) in a variety of recording modalities. The success of the artifact removal method is evaluated by comparing the residual artifacts across repeated stimulation trials and estimating neural recording SNR as well as the ARR before and after removing artifacts.

### Single-Channel Electrical Stimulation of Sciatic Nerve

Monopolar stimulus pulses (0.2 ms duration, cathodal current, 0.5 Hz stimulation rate) with 6 current amplitudes (10−320 μA, octave increments, both sub- and supra-threshold) were delivered in pseudo random order to one end of sciatic nerve with a platinum iridium electrode (Figure 1A). Evoked action potentials along with stimulation artifacts were recorded from 40 teased sciatic nerve filaments and the quality of artifact removal using the proposed linear Wiener filter was assessed under various conditions.

The artifact prediction filter accurately predicts the recorded artifacts regardless of the current amplitude delivered, as shown in Figure 1B, where a representative recording of the lidocaine treated artifacts is superimposed with the Wiener filter predicted artifact (Wiener filter estimated using currents between 10 and 320 μA under lidocaine). The prediction filter was obtained by correlating the input stimulation current with the recorded artifacts using the Wiener-Hopf Equation (Eqn. 3). This filter is subsequently used to filter the input current in order to predict the artifacts. As seen for the entire session, the recorded artifacts under lidocaine treatment (purple) are highly overlapped with and indistinguishable from the predicted artifacts (red). Likewise, magnified views of the recorded artifacts from different stimulating amplitudes are indistinguishable from the predicted artifacts as displayed in Figure 1C. The overlapping waveforms between the actual and the linear Wiener-filter predicted artifact verify the hypothesis that recording artifacts follow a linear relationship with respect to the input current signals. We explicitly tested for linearity by first plotting the relationship between input current amplitude and the peak-to-peak amplitude of recorded artifact which showed an exceptionally high correlation coefficient across all recordings (Figure 1D; r^2^ =0.9997± 0.0004, Mean ± SD, *N* = 40). Similarly, the relationship between the peak-to-peak amplitude of the recorded (with lidocaine) and the linear filter predicted artifacts was likewise highly correlated (Figure 1E; r^2^ =0.9997 ± 0.0004, Mean ± SD, *N* = 40). These results suggest that, for this experimental preparation, the recorded artifacts scale linearly with respect to the current input, such that the linear Wiener filter accurately predicts the recorded artifacts regardless of the amplitude of the stimulating current.

The artifact prediction and removal procedure accurately isolated neural responses for a range of filter estimation conditions. The artifact prediction filters were estimated using artifact recordings either from subthreshold stimulation or under lidocaine treatment (see section “Materials and Methods”). The estimated filters were then used to predict and cancel out the recording artifact during suprathreshold stimulation (pre-lidocaine at 320 μA). Figure 1F shows a representative suprathreshold recording (320 μA current stimulation; black curves) along with the predicted artifacts derived from each of the estimated Wiener filters (gray box). As a control, we also obtained artifact recordings following the application of lidocaine which blocks action potential generation so that the recorded signals consisted of pure stimulus artifacts as shown in Figure 1F (purple, top left). This post-lidocaine artifact signal was subtracted from the original recordings (pre-lidocaine at 320 μA, black) which allows us to isolate the neural response component (Figure 1F, purple, bottom left). For the Wiener filter cancelation method, we first used the artifacts evoked from highest measured subthreshold current to derive the artifact prediction filters. Using this filter, we subsequently predicted (Figure 1F, blue, top) and subtracted the predicted artifacts from the suprathreshold stimulation recordings (Figure 1F, blue, bottom). The Wiener filter obtained using subthreshold stimulation accurately predicts the recorded artifacts during suprathreshold stimulation and is able to isolate the neural activity. An advantage of this approach is that, unlike lidocaine treatment, it does not require a pharmacological treatment to block neural activity in order to isolate and remove the artifact signals. Next, we estimated the artifact prediction filters using the lidocaine treated artifacts from the lowest five current intensities (10−160 μA) and predicted the artifacts at 320 μA stimulation (green, top). Since the currents used for the filter estimation and subsequent prediction are not the same, this test serves as a cross-validation as well as assessment of linearity. As can be seen in Figure 1F (green, bottom), subtracting the predicted artifact from the recorded waveform substantially reduces the artifact size and successfully isolates the action potential. Next, we carried out the same procedure but estimated the Wiener filters using the lowest five current intensities along with the first five trials of the 320 uA lidocaine session (10−320 μA, 55 trials used, cross-validated condition, brown) or the entire lidocaine recording session (10−320 μA, all 60 trials used, red). As exemplified for each of these cancelation examples, the isolated neural signals obtained from artifact removal by the Wiener filter method (gray box, bottom) are nearly identical to the experimentally isolated neural signals using lidocaine treatment (purple, bottom).

We next quantified the artifact cancelation performance for each of the above scenarios. The cancelation performance depended on the data used to estimate the artifact prediction filter, particularly the number of artifacts and the signal-to-noise ratio of the artifacts used for filter estimation. The artifact reduction ratio (ARR, see section “Materials and Methods”) quantifies the attenuation of the artifact spectrum (in dB) following cancelation and is shown in Figure 1G for each of the conditions tested. The lowest ARR (measured between 300 and 3000 Hz) was observed for the subthreshold condition (22.8 ± 4.4 dB, Mean ± SD; *N* = 40 fibers) which is as expected due to fewer artifacts used (*N* = 10) for the estimation of prediction filter and the fact that the measured artifacts are relatively low amplitude and thus susceptible to background noise (i.e., low signal-to-noise ratio). The ARR improved to 28.1 ± 3.5 dB (Mean ± SD; *N* = 40 fibers; cross validated) when lidocaine treated artifacts from 10 to 160 μA stimulation were used to estimate the Wiener filter. The ARR further increased to 29.9 ± 4.7 dB (Mean ± SD; *N* = 40 fibers) which was calculated using the predicted artifact built from 55 trials of lidocaine data (10−320 μA) against the remaining 5 trials of 320 μA lidocaine treated artifacts (also cross-validated). The ARR increased to 39.9 ± 3.3 dB (Mean ± SD; *N* = 40 fibers) when lidocaine artifacts from all of 60 trials were used to estimate the artifact prediction filter (10−320 μA). As a reference control, we used the recorded artifacts from each trial of the lidocaine treated signals at 320 μA current stimulation to cancel the artifacts for all of the remaining trails (e.g., trial 1 artifact was used to predict trials 2−10; 2 was used to predict 1, 3−10; etc.). This control artifact removal serves as a way of assessing the inherent noise in single trials of the recorded data and also serves as a way of canceling artifacts without requiring the need to assume linearity as for the Wiener filter method. The ARR for this procedure (30.7± 6.8 dB; Mean ± SD; *N* = 40 fibers) is comparable to our cross-validated artifact removal performance (29.9 ± 4.7 dB, Mean ± SD; *N* = 40 fibers). This suggests that the Wiener filter artifact removal performance is comparable to the performance obtained using real recorded artifacts for removal. Thus, the Wiener filter cancelation performance for this example is largely limited by the intrinsic noise in the recording.

Collectively, these examples demonstrate that the Wiener filter cancelation method can achieve exceptional cancelation performance and is able to generalize by predicting and canceling artifacts across multiple amplitude conditions.

### Bilateral Cochlear Implant Stimulation

The artifact removal procedure was also tested with high-rate bilateral cochlear implant stimulation in rat while concurrently recording from a silicon array electrode implanted in the IC. In the first example, constant amplitude biphasic electrical pulse sequences were delivered at a pulse rate of 300 Hz synchronously to both ears, at different interaural delays (ranging between −160 us to +160 us, 40 us steps; see section “Materials and Methods”). An example raw recorded waveform from one IC electrode channel is shown in Figure 2A (black), along with the predicted artifact waveform (blue). For this example, the artifact prediction filters were estimated separately for each ITD condition using half of the response trials from each particular ITD. The remaining trials at a given ITD are used to test artifact removal quality (cross-validation). As can be seen for a recording segment (ITD = 0 ms), the predicted artifact signals are largely superimposed and are visually indistinguishable from the recorded artifacts on the neural recordings. Synchronized action potentials are observed immediately following the delivery of electrical stimulus current pulses. Upon subtracting the predicted artifact (blue) from the neural trace (black), the cleaned neural trace is exceptionally clean with no evident sign of stimulation artifacts and no evident sign of waveform distortions (Figures 2A,B, red). Spectral analysis of the recorded signal prior to panel (Figure 2C, black) and after artifact removal (Figure 2C, red) confirms a substantial reduction in the artifact size. The artifact spectrum has harmonic components with a 300 Hz fundamental (blue) which dominates the original recording (black). Upon removal of the predicted artifact, there is a substantial reduction in the artifact components (red). Overall, the average artifact reduction at harmonics of the stimulation frequency is 27.2 dB (between 300 and 6000 Hz; averaged across all ITD conditions; Figure 2D).

We also delivered bilateral electrical stimulation sequences containing pulse amplitudes that varied dynamically over time (see “Materials and Methods”) as shown in Figure 3. We used time-varying amplitudes and different ITDs in order to determine whether the stimulus current-artifact relationship is linear and to determine whether the Wiener filter prediction method can generalize to dynamic stimulation scenarios. For this example, the pulse train amplitudes were ramped on-and-off with a smooth window and the pulses were delivered at multiple ITDs (between −160 and +160 μs in 40 μs; pulse rate of 300 Hz; see “Materials and Methods”). The artifact prediction filters were estimated using all of the ITD and amplitude conditions (1/2 of the data for estimation and the remaining half for validation; validation data is shown) using Eqn. 3 and two artifact prediction filters were derived, one for the left ear and the other for the right ear (64 filters total; 32 recording channels × 2 filters / recording channel). These filters were then used to predict the artifact waveforms for all of the ITD conditions. As can be seen from Figure 3A for a representative recording channel, the predicted artifacts (red) derived with the two-channel Wiener filter largely overlap the recorded artifacts in the original neural recordings (black; shown at three different scales). The peak-to-peak voltage amplitudes of these artifacts are highly correlated with the delivered peak-to-peak current amplitudes (Figure 3C, r^2^ = 0.9981± 0.0001; Mean± SEM) as well as the peak-to-peak voltages of the predicted artifacts (Figure 3D, r^2^ =0.9981± 0.0001; Mean± SEM), indicating that the stimulation current and artifact follow a linear relationship. By subtracting the predicted artifacts from the original recordings, we were able to isolate action potentials from a single neuron (Figure 3B). Although there are still some artifacts visible in the cleaned recording (Figure 3B, arrows), the artifact size has been dramatically reduced (cross validated ARR = 25.0 dB, between 300 and 6000 Hz) making isolation of this single neuron possible.

### Single- and Multi-Channel Electrical Stimulation in Auditory Midbrain

We also tested the artifact removal procedure by delivering random biphasic electrical pulse sequences (Poisson distributed pulse intervals, 164 μs pulse duration, and 40 μA current amplitude, Figure 3A) to an auditory midbrain electrode while neural activity was concurrently recorded from rat auditory cortex. As can be seen in Figures 3B,C, the extracellular neural activity (Figure 3B, highpass filtered above 300 Hz) and the corresponding unfiltered recordings (Figure 3C, unfiltered) both contain stimulation artifacts that are substantially larger than the target neural signals.

We numerically estimated a digital single channel Wiener filter (*N* = 40 order; 1 stimulation x 1 recording channel) to predict and subsequently remove the electrical stimulation artifacts (see “Materials and Methods”). Figures 4B,C show the raw cortical recordings (top panels), the predicted artifacts (middle panel) and cleaned neural traces obtained by subtracting the predicted artifacts from the raw recordings. The artifact prediction algorithm accurately predicts the timing and amplitude waveform of the electrical artifacts and, upon subtraction, the procedure successfully isolates either the extracellular waveforms or low-frequency local field potentials in the neural signal. Magnified traces of the extracellular recordings (marked by ^∗^ and ^∗∗^) are presented in Figure 4D to show the cleaned neural recordings at a higher resolution. Notably, the algorithm is able to subtract the artifacts that occur in the vicinity of neural spiking with no visible signs of neural waveform distortions.

Performance metrics of the artifact prediction and subtraction algorithm for this recording is shown in Figure 3E (applied to the broadband unfiltered signal). The signal-to-noise ratio of the original recorded waveform varies with frequency but is generally in the order of −10 to −20 dB. Upon subtracting the predicted artifact, the cleaned SNR is ∼20 dB with an average SNR enhancement ranging between 30 and 45 dB (average = 39 dB between 300 and 6000 Hz). Thus, there is a marked reduction in the artifact size and, as seen in the zoomed neural recordings, there are no visible distortions created by the subtraction algorithm.

We also successfully used the artifact removal during high throughput multi-channel electrical stimulation (16 stimulation channels) of the auditory midbrain while concurrently recording with a tetrode array (4 channels; see “Materials and Methods”). In this instance, the Wiener filter was applied in matrix form (Eqn. 3), which allowed us to predict the artifacts generated by all of the stimulating channels on each individual neural recording channel (16 stimulation × 4 recording channels). Random pulse sequences (100 pulses/s) were delivered to the 16-channel auditory midbrain array (Figure 5A; 10 μA pulses delivered across four randomly chosen electrode channels simultaneously) while recording from auditory cortex electrodes. For this multi-stimulation site configuration, we numerically estimated the digital filters that predict the artifacts generated by each of the electrical stimulation channel. Filtered and unfiltered neural recordings, predicted artifacts, and the cleaned neural traces are depicted for both the filtered (Figure 5B) and unfiltered (Figure 5C) data. As for the single channel electrical stimulation, the artifact prediction filter is able to accurately predict the measured artifacts during multi-channel electrical stimulation, resulting in minimal distortion of the extracellular signals or the local field potentials. Prior to removing the artifact, the SNR for this recording dips to approximately −15 dB at ∼3 kHz (Figure 5E). Following artifact removal, the SNR hovers around ∼20 dB with an overall improvement in the range of 30−45 dB across the frequency range (average artifact reduction ratio = 33.5 dB from 300 to 6000 Hz; Figure 5E).

Finally, we assessed the linearity of the artifact-current relationship by delivering multi-channel pulse sequences of time-varying amplitude (Figure 6). In this example, the amplitude of random spatio-temporal pulses was modulated over time between 0.1 and 10 μA as illustrated in panel Figure 6A (11 logarithmic steps; color designates the current amplitude). By considering pulse sequences that contain multiple concurrent pulses of time-varying amplitude we are able to assess linearity, which requires that artifacts *scale* in amplitude and *summate* linearly with the respect to the input current signals. The multi-channel artifact prediction filters were derived for this example using the recorded data by applying Eqn. 3 and the predicted artifacts were then derived. As can be seen for two of the four recorded channels (Figures 6B,C), the procedure accurately predicts the recorded artifacts and the resulting cleaned neural traces show no evident signs of artifacts (Figures 6B,C, bottom; Figures 6D,E, magnified view). Linearity was assessed by plotting the recorded versus the predicted peak-to-peak amplitudes of the artifacts (Figure 6F). As can be seen from the scatter plot there is clustering along the diagonal. Variability along the diagonal for each cluster reflects amplitude variability created by the summation of randomly selected stimulating channels (4 out of 16 channels are stimulated concurrently). Each stimulating channel has a distinct impedance (transfer function) and hence a distinct artifact on the recorded channel with unique amplitude. Consequently, there are 16!  /(12! 4)=1820 possible channel combinations (4 choose 16) and a total of 10920 unique artifacts (1820 artifacts/amplitude × 6 amplitudes). By comparison, variability orthogonal to the diagonal reflects the variability in the neural signal of interest, which is present in the original recorded trace. As can be seen, for very small input currents (<500 μA) the artifact peak-to-peak amplitudes are smaller than the detected peak-to-peak amplitudes from artifact free neural signal segments (0.5 ms window used to detect the peak-to-peak voltage; Mean peak-to-peak voltage of artifact free segments = 100 μV, dotted blue line Figure 6D). Thus, for such small stimulation currents, the detected peak-to-peak amplitudes within the artifact measurement window are actually corrupted by the peak-to-peak amplitude of the neural signal. This neural signal variability represents measurement noise and creates a slight curvature in the scatter plot for currents below ∼ 500 μA. Despite this, the accounted artifact variance with a linear model was exceptionally high (r^2^ =0.9981± 0.0001, Mean± SEM) suggesting that the artifacts follow a linear relationship with the current input.

Overall, these examples demonstrate that a multi-channel linear prediction filter is able to account for the recorded artifacts generated via spatio-temporal summation from multiple dynamically changing current inputs.

### The Impact of Data Length on Artifact Removal Quality

**FIGURE 7:**
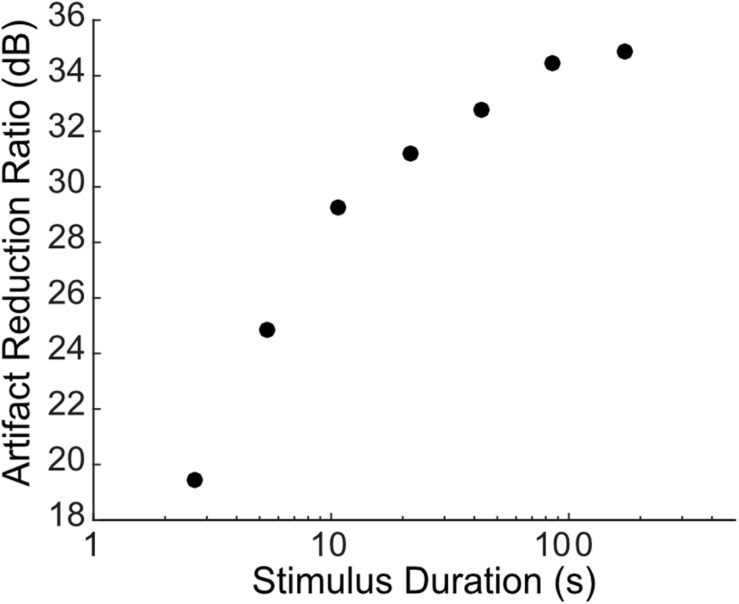
Dependence of artifact reduction quality on data length. The artifact reduction ratio (ARR, measured between 300 and 6000 Hz) is shown as a function of the data length (varied between 2.7 and 171.8 s in octave steps) used to estimate the Wiener prediction filter for the example of Figure 5. The cross-validated ARR increases with increasing data length with a net improvement of ∼2.5 dB per doubling of the data length.

As seen from different examples, there are some discrepancies in the artifact reduction ratio between the different recordings which varied between ∼25 and 40 dB for the different examples tested. This discrepancy is in part accounted by the quality of the estimated artifact prediction filters, which is expected to depend on the length of the recorded data and the number of pulses delivered. For instance, the artifact prediction filter obtained for the subthreshold sciatic nerve stimulation were derived from slow rate pulse sequences (0.5 pulses/s) of relatively short duration (10 s total) and thus relatively few artifact measurements (10 pulses total), which likely resulted in the low ARR (∼20 dB). This contrast the auditory midbrain and cortical recordings reported in Figures 4, 5, where longer sequences were used and pulses were delivered at a much higher rate (Figure 2, 300 pulses/s; Figure 3, 16 pulses/s; Figure 4, 100 pulses/s), resulting in a much higher number of artifact measurements for the filter estimation and consequently a higher ARR (∼30−40 dB). The impact of the estimation data length (or equivalently number of artifact pulses used to estimate the filters) on the quality of the algorithm is shown in Figure 7 for the auditory cortex recording of Figure 5. The recorded data was portioned into segments of a fixed duration (2.7−172 s; corresponding to ∼270−17,200 artifacts) and the filters were re-estimated using the partitioned data followed by the artifact prediction and removal procedure. As expected, the ARR improves with increasing estimation data length, or equivalently the number of artifacts used to estimate the filters, with an average improvement of ∼2.5 dB per doubling of the data length.

## Discussion

We have developed an optimal multi-channel artifact removal procedure that accurately predicts electrical stimulation artifacts using both the stimulating current signal and the estimated linear transfer function between each stimulating and neural recording electrode. The procedure is flexible and can be implemented in a wide range of applications and recording modalities, including high rate and multi-channel electrical stimulation. The procedure was validated in three different neural stimulation settings: single-channel stimulation of sciatic nerve axons, bilateral (two-channel) cochlear implant stimulation and multi-channel stimulation of the auditory midbrain, where we demonstrate a net reduction in the artifact size of 25−40 dB.

Compared with other artifact removal methods, the novelty of our approach is two-fold. First, it requires establishing linear filter coefficients that account for the transfer functions of each stimulus-recording interface, a process that needs only a modest amount of recording data (10−100 s). An added benefit is that the filter coefficients can be easily updated as needed to account for the temporal drifting of the stimulus-recording coupling, thus potentially allowing for adaptive artifact removal over a long recording periods (e.g., days to months). Second, the procedure utilizes the information of the stimulus signals that are the source of artifact and readily available in most instances, but are neglected by conventional artifact removal procedures. This allows our method to remove artifacts in neural recordings evoked by arbitrary stimulus waveforms (e.g., variable amplitudes, multiple channels etc.), which is not possible with conventional artifact removal algorithms.

Our novel artifact removal procedure capitalizes on passive linear electrical coupling of stimulus signals through tissue and air (resistive, capacitive, and/or inductive) that gives rise to the artifacts in the records (Rivnay et al., 2017). Artifacts, in this regard, correspond to electrical signals that are not neural in origin and are directly dependent on the presence of the recording electrodes and their electrical characteristics. We confirmed the underlying linearity assumption by delivering current pulses of different amplitudes and demonstrating that the artifact peak-to-peak amplitudes exhibit an exceptionally high correlation with the delivered current amplitude and/or the predicted artifacts (Figures 1D,E, 3C,D, 6F; r^2^ >0.998). Furthermore, the multi-site stimulation experiments, which successfully removed the electrical artifacts using multi-channel linear Wiener filters (Figures 3, 5, 6), suggest that electrical artifacts summate linearly thus further supporting the linearity assumption. Several prior studies have demonstrated that electrical stimulation artifacts can follow complex and non-linear relationship with the input currents (Montgomery and Jr, 2006; Mena et al., 2017), which would invalidate the use of a linear predictive filter approach as used here. One plausible explanation for this difference is that these prior studies carried out neural recordings within relatively close proximity to the stimulating electrodes. In such instances, it is highly probable that short-latency non-linear neural activity (e.g., sub-threshold pre- or post- synaptic activity) and other extracellular field potentials interfere and summate with the electrical artifacts, which likewise exhibit short latency. Such short-latency neural signals could be interpreted as artifacts even though technically they correspond to propagating activity through the neural circuitry. Although such a scenario is not evident for the recordings performed here, such short-latency neural activity could make it difficult to detect specific types of neural activity (e.g., action potentials) and could potentially limit the ability to accurately estimate the artifact prediction filters. Stimulation artifacts can also potentially exhibit a non-linear relationship if the artifact amplitudes saturate the recording amplifiers or if they exceed the voltage limits of the digital-to-analog converter. This in itself is not a limitation of our technique and could be circumvented through the use of appropriately selected neural recording hardware.

As demonstrated, the Wiener filter approach can accurately predict and remove recording artifacts in a variety of stimulation settings including single- and multi-channel stimulation, high rate stimulation, as well as stimulation with time-varying amplitude and/or shape. Conventional procedures based on template subtraction are often not able to eliminate artifacts in these settings because finding a template that matches the shape of all artifact waveforms is not always possible (Wichmann, 2000; Hashimoto et al., 2002; Qian et al., 2017). This is especially true when the electrical stimulation currents consist of variable amplitudes and shapes or when multiple current pulses from a single or multiple channels summate over time. However, we note that for simple stimulation scenarios with temporally isolated non-overlapping artifacts, template cancelation should produce similar results as our method because templates are derived using event triggered averaging, which for such scenarios is equivalent to generating a Wiener filter (de Boer and Kuyper, 1968). Other established artifact removal procedures utilizing independent component analysis (Makeig et al., 1996; Vigário, 1997; Gilley et al., 2006; LeVan et al., 2006; Debener et al., 2008; Rogasch et al., 2014) assume independence between neural activity and artifact sources, which is not always satisfied at suprathreshold stimulation condition that evokes synchronized neural activity. Thus, some of the estimated artifact components can contain both neural activity and artifacts, which can distort and eliminate relevant neural signals. Recently, a statistical model-based artifact cancelation procedure was developed to successfully remove artifacts from multiple sources to enhance spike sorting of recorded neural data (Mena et al., 2017). This statistical model-based approach is advantageous when the current inputs are not known, however, the procedure assumes that artifacts on a given channel are relatively stable and the procedure is not designed to account for dynamically varying artifacts.

By numerically estimating the linear transformation between each stimulation and recording channel and accounting for the input current waveforms, our procedure is able to generalize and accurately predict artifacts that dynamically vary over time. Linear models have been previously applied successfully to predict and remove artifacts from cortical recordings (Trebaul et al., 2016). The linear removal procedure assumed that the artifact transfer function can be accurately described by a first-order capacitive and resistive circuit. Our method extends on such an approach by providing a more general framework that is applicable across vastly more complex stimulation scenarios. First, although the use of a circuit based model provides a first order approximation of the artifact transformation, it cannot account for multiple signal transmission paths that may be present, such as simultaneous conduction through the neural tissue and through air medium (inductive coupling). The use of Wiener filters allows us to empirically measure the transfer function of each stimulating-recording pair which can theoretically account for such scenarios. Furthermore, as shown for various examples, our procedures is also able to generalize across a variety of complex stimulation conditions including multiple inputs, multiple outputs, variable current amplitudes, and multiple stimulation delays such as for the cochlear implant and auditory midbrain stimulation examples. The flexibility of our approach is exemplified in the dynamic multi-channel stimulation example (Figure 6), where randomly selected inputs of different amplitudes were activated creating a total of 10920 possible distinct artifacts. Despite this, the linear Wiener filter accurately predicts and cancels the incoming artifacts even for this complex scenario. As far as we are aware of, there are presently no artifact cancelation procedures available that can handle this high variability since all the available procedures require relatively stable artifacts over time.

The artifact reduction ratio varied between ∼25 and 40 dB for different recording modalities tested. Differences between the different modalities are due to the available data used for deriving the filter coefficients and the intrinsic SNR of the data itself. As demonstrated for the sciatic nerve recordings, the quality of the artifact removal is limited both by the data length and number of artifacts in the training data. The ARR for the sciatic nerve recoding is ∼22 dB when the filters are estimated using subthreshold activity which only contains 10 artifacts and the artifacts themselves are relatively small in relationship to the neural activity (low SNR for estimating the artifact). Prediction quality and hence the artifact removal effectiveness improves dramatically when artifacts from higher current amplitudes are used to estimate the filter coefficients. This improvement occurs, in part, because substantially more artifacts are used to estimate the filter coefficients (increase in data size) and because the measured artifacts for high current amplitudes have a higher SNR. Similar results are observed for the multi-site stimulation scenario, where the quality of the artifact removal improves upon adding more data to the filter estimates (Figure 7). For this scenario, we note that the increase of SNR for every doubling of the data is ∼2.5 dB, which is close to the theoretically expected value under the assumption that measurement noise is independent (Marmarelis and Marmarelis, 1978) (3 dB improvement per doubling of the data length; i.e., estimation error variance decreases inversely proportional to data length).

Because of the computational efficiency of the linear Wiener filter algorithm, the proposed artifact removal procedure has potential applications for real-time assessment of neural function and real-time feedback control (Wilson et al., 1991; Schachter and Saper, 1998; Dhillon and Horch, 2005; Lebedev and Nicolelis, 2006; O’Doherty et al., 2011; Mc Laughlin et al., 2012; Hartmann et al., 2014). On the one hand, the artifact removal filters can be estimated with a dedicated segment of recorded data. During such a period artifacts cannot be removed and the acquired data is strictly used for training the artifact removal filters. The speed of the subsequent artifact removal will be limited by the recording hardware delays which can be less than a few milliseconds with appropriately selected hardware and which are sufficiently short for most feedback applications. Alternately, Wiener filter coefficients can be estimated and implemented iteratively using solutions that update the coefficients as needed (Hayes, 1996), however, this approach would require additional computing resources to iteratively estimate the filters with the incoming data. Such an adaptive approach can potentially account for the drifting of the stimulus-recording that will be investigated in a future study. In theory, it allows the filter to be updated and optimized at any time by introducing new training data or by continuously using the recorded data itself to estimate the filter coefficients in real-time. Such iterative implementations would also allow for quantitative estimation of the stimulus-recording conditions over time, which may exhibit non-stationary behaviors for chronic recordings (e.g., due to changing electrode impedance over days or movement of electrodes, etc.).

Overall, the proposed Wiener filter artifact prediction and removal procedure has the potential for a broad range of applications requiring concurrent neural stimulation and neural recording from multiple channels. Wiener filter estimation and prediction approaches have a long history and are well established (Wiener, 1949; Hayes, 1996). They are computationally efficient requiring little data to estimate the filter coefficients (10−100 s to achieve 25−40 dB ARR in our examples) and do not require specialized hardware. Hence, the approach can be easily adapted for real-time applications and applications requiring real-time assessment of neural function and behavior.

## Data Availability Statement

The datasets generated for this study are available on request to the corresponding author.

## Ethics Statement

The sciatic nerve and auditory midbrain stimulation studies were reviewed and approved by the University of Connecticut Institutional Animal Care and Use Committee and follow American Veterinary Medical Association guidelines. Cochlear implant stimulation studies were approved by the Department of Health of Hong Kong (#16-52 DH/HA&P/8/2/5) and the Animal Research Ethics Subcommittee of City University, Hong Kong.

## Author Contributions

MS, KD, AN, HR, and ME acquired the auditory midbrain and cortical data. MS analyzed the auditory midbrain and cortical data. LC and BF acquired and analyzed the sciatic nerve data. JS and NR-K acquired the cochlear implant data. JS and ME analyzed the cochlear implant data. ME, MS, and AN developed the filter prediction and subtraction algorithm. MS, ME, LC, and BF wrote the original manuscript. All authors contributed to editing of the subsequent versions.

## Conflict of Interest

HR has ownership interest in Elemind Technologies, Inc., and this private company did not sponsor this research. The remaining authors declare that the research was conducted in the absence of any commercial or financial relationships that could be construed as a potential conflict of interest.
